# Girls and Gangs: A Decade on From the Firmin Report and What Has Changed?

**DOI:** 10.1177/10778012241233005

**Published:** 2024-02-14

**Authors:** Deborah Jump, Rachel Horan

**Affiliations:** 1 5289 Manchester Metropolitan University, Manchester, UK; 2 The Averment Group, Manchester, UK

**Keywords:** gangs, gender, violence, girls, women

## Abstract

Presenting data from the first phase of a U.K.-based 5-year mixed-methods study, we restart a decade-long conversation into Girls and Gangs and Violence Against Women and Girls (VAWG). The relationship between the two is not mutually exclusive and coupled with the recent optics surrounding youth violence and gendered violence, we discuss how the needs of women are being somewhat hindered as a result of U.K. governmental vacillation. We therefore consider the serious impact of VAWG and the concomitancy with youth violence/gangs. By drawing on contemporary feminist criminological theorizing, we aim to galvanize governmental responses to prioritize the needs of women at a time when policymakers are arguably poised to listen.

## Introduction

This article considers the research and policy landscape a decade on from Carleen Firmin's nascent work into *Girls and Gangs* published in 2009. It presents the changing discourse of girls and young women (G&YW)'s involvement with gangs and serious youth violence (SYV therein) with data from a 5-year mixed-methods research study into the impact of SYV on G&YW aged 14–24 years in the Northwest of England. By presenting our emerging findings we are picking up the conversation. More than 10 years later, we ask questions of governments and policymakers who are only now starting to recognize the links between adverse childhood events and trauma, and the trajectory created for mental health outcomes and opportunities for young women to flourish. This article discusses a different future concerning young women's mental health outcomes as a result of early identification by trusted adults and professional services. We purport that over the past decade, the landscape of Violence Against Women and Girls (VAWG) has come into clearer focus, but much more work needs to be done that considers the nuances of young women's and girl's vulnerability to exploitation and abuse, and how this could potentially confabulate with mental health measures and childhood trauma.

## Girls and Gangs: The Historical Picture

Between 2008 and 2011, the Female Voice in Violence research project was established between ROTA (Race on the Agenda) and the Women's Resource Centre to explore the relationship between SYV and gangs and the impact this has on G&YW (Firmin, 2009). Promises were made to seriously highlight the experiences of young women affected by SYV and gangs, as well as highlighting best practices among services working in this burgeoning area. A series of high-level recommendations were made to illuminate the experiences of young women, and with that, a push for meaningful change among policy and practice. The most salient recommendation being: “placing offending within a context that includes women is crucial, as any other context fails to depict the reality of what is taking place” (Firmin, 2009, p. 15).

With the watershed ROTA report, *This Is It This Is My Life* in 2011, Firmin argued that the impact of SYV- and gang-related violence is a child protection issue and therefore should be recognized as such in both policy and practice. Moreover, the subsequent planning and policy ratification needed at both governmental and local levels was lacking and needed an overhaul to consider VAWG as a serious issue, and with that, funding to design and develop specialist provision that meets the needs of vulnerable young women. This provision needed to be specifically tailored to include prevention, identification, intervention, and exit from harmful scenarios from young women embroiled in the maelstrom of violence.

Clear definition of harm is also crucial here. Tara Young (2009) made this case clearly when she argued that the definition of the “Shemale Gangster” is flawed and needs further consideration if we are to ameliorate the harms caused by male violence. Young suggested that “gang research in the UK is quintessentially male; it is dominated by male researchers who study the criminal behaviour of other men” (Young, 2009, p. 235), and to make any strides in policy change or ways of thinking about this issue, we needed to move away from the stereotypical image of young women as ancillary sexualized bodies servicing the gangs as mere victims of male power and violence.

Young (2009) and Batchelor (2005) argued that before considering interventions with “gang-involved” young women we need to locate the concept of their identity in a personal and autonomous context, therefore acknowledging the reality of their lives as a potential response to the disempowerment and lack of respect they feel as young people in their communities. This is while simultaneously recognizing that the coalition of peers in a group (both male and female) may be a further response and direct need to garner the psychosocial support that could be lacking in their lives (Young, 2009). All this considered, the framework for understanding young women's involvement in SYV and “gangs” should not be dislocated from the social context in which it resides. Put simply: “engagement in violent behaviour should be based on the appreciation of the normalisation of threat and violence within their life trajectories; as an attempt by young women to forge friendships and exert some control over their lives, and a way to stave off further victimisation” (Young, 2009, p. 235).

To return to Firmin, the recognition of the complexity and relevance of social context is not lost. Firmin (2015) and Firmin and Lloyd (2022) have developed “contextual safeguarding,” a social care innovation that responds to extra-familial harm faced by children within communities, peer groups, and contexts. Four features of a contextual safeguarding response work to address extra-familial risk (i) target the contexts in which harm/abuse occur; (ii) use child welfare and child protection as the principal focus and legislative framework; (iii) feature partnerships with individuals/organizations who have a reach into, or responsibility for, the places where harm has occurred; and (iv) measure the contextual impact/outcomes of the response (Firmin et al., 2016).

Holmes and Smale (2018) have also continued to progress an innovative approach with the introduction of the concept of “transitional safeguarding.” It is an “approach to safeguarding adolescents and young adults fluidly across developmental stages which builds on the best available evidence, learns from both children's and adult safeguarding practice and which prepares young people for their adult lives.” It recognizes that transition is a journey, not an event, and that every young person will experience their journey differently. This overarching approach is designed to respond to local context (Cocker et al., 2021) and it again, stretches out statutory and professional response beyond individuals and families to respond holistically.

## Girls and Gangs: Where Are We Now?

The problem of an effective and relevant response to the dynamic challenge of SYV, gangs, and group offending continues long after the original theoretical and policy debates of the mid-2000s. Yet, after years of decline, serious violence and violent crime began to rise in 2014; a more recent trend that included a rise in offenses involving knives and firearms and shifts toward younger victims and perpetrators (HM Government, 2018). The need for change in both understanding and approach was obvious, and arguably, still is. A Serious Violence Strategy was launched by the U.K. Government (HM Government, 2018) and this was concomitant with a much-needed reframing of perspectives, least of all, an integrated response that recognizes the vulnerability and safeguarding of young people. This targeted safeguarding and child protection response (HM Government, 2021) together with recognition of the prevalence of mental health issues drawing young people into SYV further galvanized our response (Jump & Horan, 2021).

Outside of policy, an acknowledgment of challenges faced by girls involved and affected by gangs and SYV continues to develop with contributions including Young (2009) as well as Khan et al. (2013). However, an accompanying holistic understanding is still not clear. The plight of G&YW associated with gangs is described by the Centre for Social Justice in its Girls and Gang paper and summarized with a searing conclusion that “one of the most concerning aspects of girls in gangs is how little we really know.” This is concerning considering it was some 7 years on from the high-profile recommendations made by Firmin in 2011. Yet again, women and girls are marginalized at the fringes of criminological discourse and policy.

This article is therefore a timely opportunity to contribute to the ongoing debate by disseminating our own findings and learning more than a decade on. By describing one of the U.K. “I Define Me” (IDM) projects, referred to as “Getting out for Good” (GOFG), a project which was overseen by Manchester Metropolitan University and led by the authors of this article, we explore whether we are any further on in addressing the harms caused to females as a result of serious youth and community violence.

## GOFG: A Global Research Approach

In 2016, the charity Comic Relief awarded 3-year funding to a number of international projects in the United Kingdom, South Africa, and Colombia. This global program, entitled IDM focused on supporting G&YW affected by gangs and those vulnerable to or being criminally exploited by gangs. Projects in the United Kingdom were evaluated in 2019, and as a result, funding was continued for an additional 2 years. This unique approach enabled these projects to develop gender-responsive interventions and respond while informed by significant contemporary learning.

We will briefly describe the parameters of GOFG, discuss some of the evidence used to design the project and its theory of change (ToC), and detail its onward evaluation, learning, and development (Jump & Horan, 2021), we will focus on the most important learning that has emerged from the project and the dominance of presenting emotional and mental health needs of the G&YW. We will then describe how the project developed its focus beyond gangs to a wider understanding of mental health, childhood trauma, and sexual exploitation, in many ways paralleling the developing evidence base and policy response. Most importantly, it will tell the stories of some of the G&YW involved in GOFG, in their own words, and how the GOFG project molded to their individual and unique stories.

### GOFG: What We Hoped to Achieve

At the start of the project in January 2017, GOFG was designed to engage with young women who were at risk of or involved in SYV and “gangs.” Our first hurdle was the ongoing complexity of defining gangs, a contentious issue that needed to be explored in order to identify inclusion and exclusion criteria to enable us to design an effective intervention. Indeed, there are numerous definitions and typologies of what defines a “gang” and reaching a consensus has perennially proved difficult (Hallsworth & Young, 2008; Ralphs et al., 2009). The 2010 Government report “Safeguarding Children and Young People Who May Be Affected by Gang Activity” distinguishes between:

- “Peer Group”—a relatively small and transient social grouping that may or may not describe themselves as a gang depending on the context.
- “Street Gang”—groups of young people who see themselves (and are seen by others) as a discernible group for whom crime and violence is integral to the group's identity.
- “Organized Criminal Gangs”—a group of individuals for whom involvement in crime is for personal gain (financial or otherwise). Crime is usually their “occupation.”

However, this above definition is also not without critique, as the relationship between the three groups is too complex and interwoven to presume such accurate categorization. Times have changed as has the context for young women. Following on from Young's (2009) earlier arguments, those definitions do not allow for young people's self-definition and identity to formulate what membership looks like for them as a distinct group, thus negating any pull factors that might be present in the narratives of young people who are drawn to this lifestyle. As previously mentioned, Firmin (2009) argued that the male-dominated account of gang violence threatened to delay long-term progress in making change, with Batchelor (2009) further arguing that regardless of whether young women are defined as “gang” members or not, some young women clearly spend vast amounts of time hanging around on the streets or going missing from home, which has important implications for their lives and their ability to flourish. What is missing here, as Batchelor (2009, p. 408) rightly points out is that “spending time with friends is a prime social activity for most young people, but girls in particular commonly describe their friendships as ‘the most important thing.’” This is particularly pronounced with G&YW such as those in projects like GOFG as young women coming from backgrounds characterized by disruption, abuse, and neglect, enables their peer group to take on heightened significance as a source of identity, approval, support, and protection (Joe & Chesney-Lind, 1995; Miller & Decker, 2001). As McRobbie and Garber (1991, p. 11) also suggested “The important question might not be the absence or presence of girls in male subcultures, but the ways in which young girls interact among themselves and with each other to form a distinctive subculture of their own.”

Considering the above and building on prior research, the Comic Relief IDM focus moved away from a previous reliance on discourse on male gang members as their source of information about females, to enable GOFG to be conducted through a more gendered lens of G&YW. We recognized early on that efforts to prevent or address gang association among females clearly needed to be “gender-specific” (CSJ, 2018).

Specific background evidence was drawn from the 2013 Centre for Mental Health report “A Need to Belong: What Leads Girls to Join Gangs” (Khan et al., 2013). In this comprehensive review of international literature on girls involved in gangs and an analysis of data collected for more than 8,000 young people, they identified a wide range of risk factors for females to become members of gangs. These include:

- Severe childhood behavioral problems and mental ill health.
- Poor maternal mental health, exposure to violence in the home, and experience of trauma.
- Low academic aspiration and disengagement with school.
- Association with antisocial or gang-involved peers and peer rejection or victimization.
- Feeling unsafe or marginalized in their neighborhood.
- High-income inequalities and social influences that devalue female roles.

This evidence allowed us to avoid getting tangled in a “gang definition” and think about gender-specific risk factors as parameters of inclusion and referral criteria. We were mindful of the age range and varying transitions of vulnerable G&YW, both in statutory involvement and also within their own stories how the project could best provide support. In addition, we identified that projects which empower both young women (and indeed men) to break the cycle of power and control in relationships is essential to supporting young people away from gang life (Eshalomi, 2020). The Centre for Mental Health (Khan et al., 2013) report also identified how preventive measures need to tackle multiple risk factors, for example, to support secure attachment in early years, to reduce maltreatment and neglect, to promote positive parenting techniques, to strengthen girls’ self-esteem and to respond quickly to the first signs of mental ill health among children. It also identifies how programs working with gang members need to be sensitive to the specific requirements of young women, for example, to foster respectful, collaborative, and empowering relationships to strengthen self-esteem, to provide safe housing, and to offer positive female role models.

Accordingly, the GOFG project adopted an approach that combined gender-specific mentoring with sporting and cultural activities for the G&YW aged between 14 and 24 years referred into the project. Together with cultural and art activities, we specifically chose sport and mentoring as an intervention after consultation with the young women and local stakeholders already working within the area where the project was based.

Sport can effectively engage young people on the verge and/or at risk of offending, by providing diversionary activities when they otherwise may be involved in anti-social behavior. The evidence for schemes such as these is also well documented elsewhere, especially regarding sport's ability to engage young people at crucial times, dismantle negative peer groups, and provide non-conventional classroom-based education to those who are currently disengaged (McMahon & Belur, 2013). Accordingly, the project team in consultation with the girls felt that sport was a good opportunity to address some of the issues they faced, least of all an opportunity to develop group-based activities and earn qualifications without the pressures of more traditional classroom-based activities. While not perfect, the combination of football, boxing, and cultural activities such as filmmaking worked well in initially engaging the young women. Once ensconced within the project young women could choose to opt out of specific activities. Indeed, football was dropped as an activity in Phase 2 of GOFG and replaced with trampolining as the young people felt more motivated toward this activity.

We took this approach as prior evidence suggests that in community and local settings sport has been effective in attracting young people and improving performance in activities in which they are not normally motivated to engage (Nichols, 2007). Moreover, this method of active learning commonly seen in sports has been identified as a key element in the “what works” literature on reducing offending (Meek, 2018). Sport, therefore, is a valuable resource in motivating young people who are both marginalized and reluctant to engage in conventional positive activities. Boxing was chosen by the young women as a sport they would like to try and thus proved to be the most enduring of all the sports activities. When delivered correctly evidence has shown that boxing can be effective in engaging people who may be presenting with anxiety and/or mental health issues (Jump, 2020). Important to this particular research design was prior data collected from Van Ingen (2021) and Gammage et al. (2022) who have also suggested that boxing can be beneficial for female survivors of domestic and sexual violence. Notwithstanding, the evidence for reduction of criminogenic attitudes and behavior when participating in boxing is weak (Jump & Smithson, 2020).

All this considered, GOFG delivered gender-specific sporting activity in the form of boxing and football and coupled with mentoring in a bespoke gender-specific 10- to 12-week program. By working closely with the mentors, we enhanced the sporting activities for the young women with the intention of contributing toward resilience building, enhancing personal aspirations, facilitating teamwork, and fostering positive peer networks, while simultaneously up-skilling the G&YW for the job market with nationally recognized assessment and qualification alliance qualifications awarded by the university.

As part of this bespoke program, the GOFG team took a participatory approach to working with young people (Smithson et al., 2021) and enabled the young people to use their voice in the research design as well as the content of the intervention. This was particularly important, as prior research has suggested that amplifying the voices of young women normally marginalized in research surrounding gangs and violence can actually empower females, rather than render them as passive participants and ancillary to male-dominated versions of hegemonic masculinity (Petterson & Karlsson, 2005).

Participatory methods are grounded in the democratization of the research process, centralizing the lived experiences of the co-researchers and breaking down hierarchies and social injustices. With this in mind, GOFG mobilized the young people to participate in the research process. Indeed, this process required a joint approach where young women felt empowered as well as feeling safe enough to express their vulnerability, and in some cases, their reluctance to change. The GOFG project aimed to raise aspirations, increase social capital, and help young women become more physically fit through a process that empowered them and included them at every level (Jump & Horan, 2021).

### GOFG: Redefining What “Out” Means

Throughout the project and the subsequent research, it became apparent quite early on that the young women involved in the program were not “gang members” or affiliated with any version of a gang typology as suggested by the 2010 governmental definitions. What was clearly identified buttressed the findings of the Centre for Mental Health report (Khan et al., 2013) which took a much more gender-specific approach whereby vulnerabilities such as adverse childhood events and trauma, living in poverty, and mental ill health, as well as low academic aspiration and disengagement with school. From the qualitative and quantitative evidence that we were able to gather, we can see that the young women referred to the program came with a high level of presenting need and this need fluctuated throughout the project. Nonetheless, the “getting out” was enmeshed with wider psychosocial factors that proved difficult to untangle and ameliorate within the project timeframe, especially given the COVID-19 pandemic throughout Phase 2 (2020–2022) of the project.

GOFG responded to the multiple systemic vulnerabilities that women and girls face, including living in residential care; a history of abuse or neglect; experience of loss; low self-esteem; learning disabilities or poor mental health; living in a gang neighborhood; or lacking friends of the same age. The G&YW who were referred to the project are given an intensive 3-month program of mentoring, advice, and activities by a local charity together with local sport, art, and cultural providers. With a focus on boxing and mentoring supported by local providers and charities, the G&YW help themselves and their peers to address pathways into and out of potential gang involvement and exploitation by devising their own solutions through up-skilling, resilience building, and peer mentorship. This mentoring relationship was key to the noted success of the project and will be discussed further in the results section below.

The main referral route was via local agencies working with G&YW who were identified as “at risk.” The “at risk” criteria remained broad to allow for those on the periphery of SYV, exploitation, and harm to be identified early. Nonetheless, young women who could be described as more entrenched were also referred to GOFG, and with carefully considered risk management procedures in place including inter-agency information sharing agreements and inter-agency referral forms that included risk identification and management approaches, young women were assigned a mentor and access to activities. The target participants were females aged 14–24 years who had been identified as being at significant risk of harm or had been involved in harmful behaviors prior to referral. The key stakeholders and referral agencies compromised of Education; Social Care & Safeguarding teams (including Missing from Home teams); Youth Justice; and Looked after Children. Importantly, in some instances, GOFG received peer or self-referrals into the project highlighting the engagement and investment of G&YW with GOFG. Between the period 2017–2021, GOFG received over 200 referrals into the project and engaged with 130 young women on a regular basis. This engagement was based on attendance at sports sessions and mentoring sessions.

### Evaluation Approach

During the 5-year GOFG project (Phase 1 [2017–2020] and Phase 2 [2020–2021]) a mixed-methods design approach was employed throughout. Activities were adjusted based on received feedback, with maximized boxing and mentoring content. Contribution analysis was undertaken, and the project ToC was collaboratively refined through a series of workshops between the research and project delivery teams.^1^

### Quantitative Methods

A Strengths and Difficulties Questionnaire (SDQ) (Goodman et al., 2000) was completed at the start and end of G&YW's involvement with GOFG along with a Satisfaction with Life Scale (SWLS) (Kobau et al., 2010), a Short Warwick-Edinburgh Mental Well-Being Scale (SWEMWBS) (NHS Health Scotland, University of Warwick and University of Edinburgh, 2008) and a MOS Social Support Survey Instrument (MOSSSI) (RAND, 2020).

A descriptive analysis was conducted to explore the presenting needs of the GOFG cohort and compare baseline assessment scores to follow-up assessments.

### Qualitative Methods

The McAdams Life Story Interview (McAdams, 2008) was reviewed and adapted for the purpose of evaluation. Each case study (*n* = 8) was undertaken individually with young people and considered whether GOFG responded to each individual and whether it had any impact on G&YW's internalized and evolving narrative. Co-produced case studies and accompanying visuals were completed.

## Results

### The Changing Tide and Challenges of the Work

The following tables summarize the quantitative data gathered during the first phase evaluation of GOFG. It conveys the high levels of presenting need and insight as to what areas of presenting need changed over the duration of G&YW's time with GOFG during this phase of the project.

Table 1 highlights the presenting needs of the G&YW at the start of their involvement with GOFG by comparing the scores of the GOFG cohort compared to national average data. As can be observed, the GOFG has higher scores on all subscales (including the pro-social scale which has a reverse positive direction).

**Table 1.** GOFG Young People SDQ Time 1 Mean Scores Compared to National Average Scores.

SDQ subscales	Possible range	Clinically significant range	GOFG			National average	
			*N*	*M*	*SD*	*M*	*SD*
Emotional problems scale	0–10	≥6	29	6.45	2.08	3	2.1
Conduct problems scale	0–10	≥5	36	3.86	1.57	2	1.6
Hyperactivity scale	0–10	≥7	36	6.08	1.50	3.6	2.2
Peer problems scale	0–10	≥4	36	3.92	1.56	1.4	1.4
Prosocial scale	0–40	≤5	36	7.86	1.87	8.5	1.4
Total difficulties score	0–10	≥18	27	20.56	4.22	10	5.3
Impact	0–10	≥2	14	2.80	1.96	2.36	1.51

*Note.* GOFG = Getting out for Good; SDQ = Strengths and Difficulties Questionnaire.

As can be observed in the above data (Table 2), the mean total difficulty score decreases over time and there are variations in subscales at each measurement point.

**Table 2.** GOFG Young People SDQ Mean Scores.

SDQ subscales	Time 1			Time 2			Time 3		
	*N*	*M*	*SD*	*N*	*M*	*SD*	*N*	*M*	*SD*
Emotional problems scale	29	6.45	2.08	15	5.20	2.21	2	6.50	3.54
Conduct problems scale	36	3.86	1.57	15	3.60	1.06	2	2.00	1.41
Hyperactivity scale	36	6.08	1.50	15	5.87	1.64	2	7.00	1.41
Peer problems scale	36	3.92	1.56	14	5.64	1.34	2	4.00	1.47
Prosocial scale	36	7.86	1.87	15	7.33	1.84	2	7.00	2.83
Total difficulties score	27	20.56	4.22	14	20.87	3.20	2	19.50	7.78
Impact	14	2.80	1.96	14	3.55	1.52	3	2.33	1.15

*Note.* GOFG = Getting out for Good; SDQ = Strengths and Difficulties Questionnaire.

Table 3 highlights increasing levels of needs when comparing Cohorts 2, 3, and 4.

**Table 3.** GOFG SDQ Scores Across Cohorts.

SDQ scale	Cohort 2 mean	Cohort 3 mean	Cohort 4 mean
Total difficulties	19.18	21.00	21.73
Emotional problems scale	5.91	5.86	7.36
Conduct problems scale	3.71	4.00	3.92
Hyperactivity scale	6.14	6.00	6.08
Peer problems scale	3.21	4.67	4.15
Prosocial scale	7.86	7.78	7.92
Impact	2.43	2.67	2.83

*Note.* GOFG = Getting out for Good; SDQ = Strengths and Difficulties Questionnaire.

In Table 4, it can be observed that SWLS scores rise in a positive direction. There is a reduction in total SWEMWBS scores when Time 1 is compared to Time 3 but an improvement in total scores at Time 2. The MOSSSI subscale also moves in a small but positive direction over Times 1, 2, and 3.

**Table 4.** GOFG Young People Mean Assessment Scores.

Scale	Time 1			Time 2			Time 3		
	*N*	*M*	*SD*	*N*	*M*	*SD*	*N*	*M*	*SD*
SWLS	23	19.35	7.52	10	24.08	7.06	5	27.60	3.91
SWEMWBS	21	21.19	6.23	12	25.67	6.02	3	24.33	5.03
MOSSSI	24	3.25	1.00	11	3.74	1.09	6	4.00	0.80

*Note.* The table displays participant's SWLS, SWEMWBS, and MOSSSI total scores at each time point. GOFG = Getting out for Good; SWLS = Satisfaction with Life Scale; SWEMWBS = Short Warwick-Edinburgh Mental Well-Being Scale; MOSSSI = MOS Social Support Survey Instrument.

In summary, data were analyzed descriptively due to the small sample size, especially at Times 2 and 3. An explanatory approach to analysis was especially relevant given the participatory approach of GOFG. We inevitably observed that the majority (78%) of G&YW started the project with high or very high levels of need.

When we analyzed the SDQ data over time, we observed that there was a small increase in the overall total difficulties scores of G&YW attending GOFG. However, we looked in much greater detail at the subscales in order to best understand this change and observed that the subscales of “emotional problems” and “conduct problems” improved over time (i.e., scores reduced), scores on the “prosocial scale” increased, which also reflected improvement. It was the hyperactivity and peer problem scales that deteriorated over the time of the project during Phase 2, with scores on the “hyperactivity” scale deteriorating the most. Again, none of these findings were statistically significant.

The qualitative data told the story of GOFG by the G&YW (Jump & Horan, 2021) and complemented the quantitative data by providing further depth to our analysis of the girl's presenting needs and issues. More importantly, it allowed young women to recount their stories in line with our research design being fundamentally underpinned by participatory methods, democratic action, and feminist theorizing. The project team felt it imperative that we captured the G&YW authentic voice and provide a space for them to recount their experience in their own words, rather than purely capturing their needs via standardized psychometric assessment batteries.

Life story interviews consistently highlighted both enduring and reactive mental health needs. Trauma, adverse childhood experiences (ACE), mental health challenges, and association with negative peer groups were frequent low points of the G&YW's life stories together with COVID and the pandemic. G&YW recounted turning points in their life stories, often occurring because of a culmination or a peak of one or several ACEs which led to a search for additional support, frequently by parents but also by involved professionals. GOFG and its acceptance criteria fitted with the presenting needs of the G&YW. The G&YW felt that the GOFG project was relevant to them, and they experienced it as reliable and engaging. The successful parts of GOFG were often considered to be their mentoring relationship, which was universally described as helpful and meaningful, therefore facilitating change and empowering the G&YW to make changes in their lives.

We argue that secure and positive attachments to mentors played a key part in the success of our findings. This particular finding aligns with Ansbro's (2022) recent work on the impact of positive attachments on probation practice. We therefore suggest that there are transposable lessons to be learned for youth offending and social care in her work. Ansbro purports that more work needs to be done to consider the theorizing around attachment theory (Bowlby, 1988) and recommends that while there are no formulaic predictions, it is equally important to recognize the impact that fractured early relationships can have on the ability for “mentalization” (Fonagy & Target, 2005),^2^ especially among those caught in the web of criminal justice agencies. Ansbro (2022, p. 10) suggests that “it is the idea that supervision offers an opportunity for people on probation to ‘feel felt’ i.e., to be mentalized, and to be invited to articulate their own and other's mental states, thus developing the skills of self-management and empathy.” The point here is that considering the mentoring relationship was key to the project, we postulate that some of the successes witnessed were as a result of another individual recognizing, tolerating, and soothing difficult emotions that can be internalized by the G&YW as a strategy for managing their own internal state, therefore enabling the growth of the “agentive self” (Fonagy, 2004). Additionally, there is also scope here for the idea that the mentoring relationship may have provided a mirroring of a painful experience—a “lived experience”—for young people to connect with, especially considering that the mentors themselves had personal experience of a variety of issues that the G&YW felt that they were currently facing. For example, one mentor had lived experience of residential care as a teenager, and another had previous personal experiences of school exclusion, bullying, and sexual exploitation.

As a result of the mentoring combined with the interventions the reported outcomes for the G&YW included mental positivity and more positive agentic perspectives. Practical and tangible outcomes were also important, such as making new friends, going to school, doing GOFG activities, and signposted activities. Feelings of hopefulness, safety, and support were all frequent to life story interviews, and from these qualitative findings, it is clear the practical outcomes also helped the young women in their positive outlooks for the future. The G&YW universally experienced benefits from GOFG and its facilitative approach that had helped them to identify and work toward their future goals and aspirations. Goals were often bigger than they had been prior to their involvement in GOFG, becoming both realistic and aspirational.

**Figure 1.**
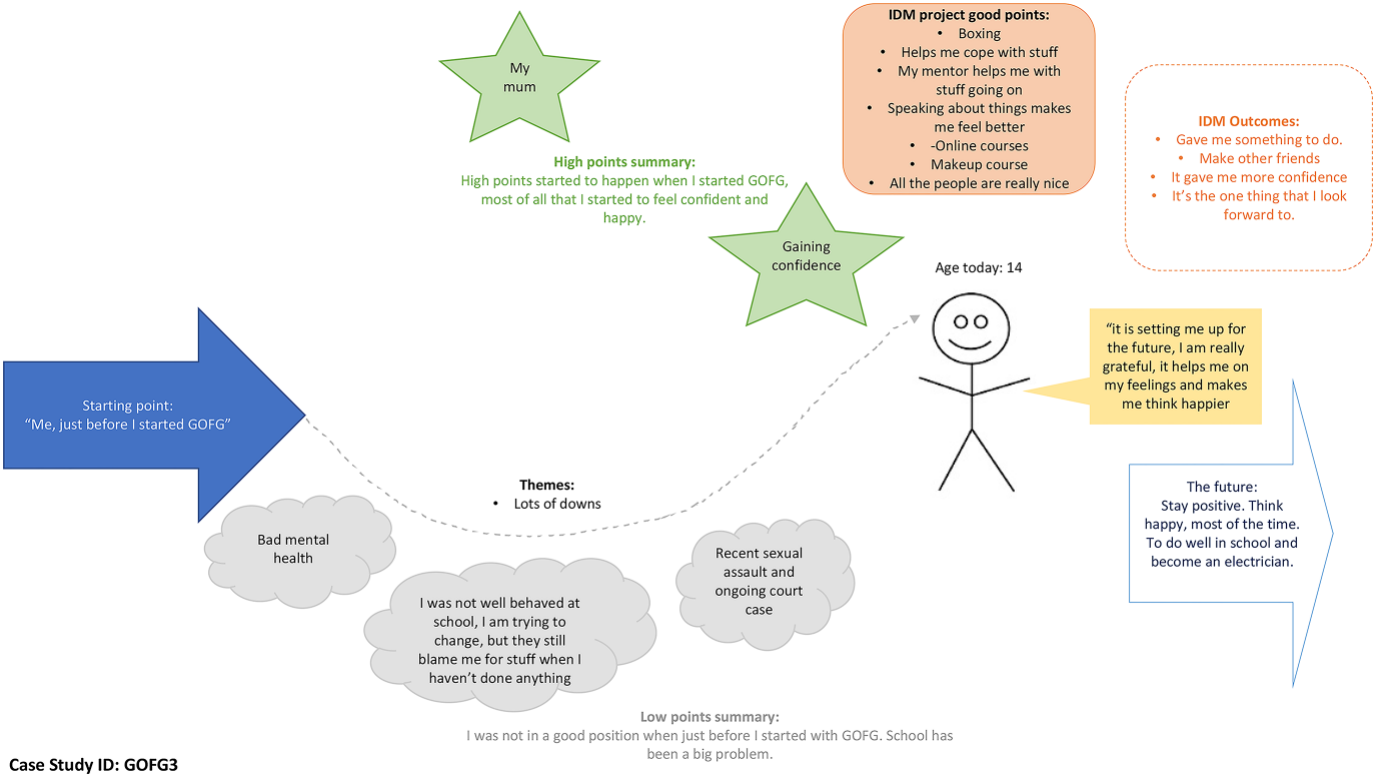
GOFG Case Study 3.

*Note.* GOFG = Getting out for Good.

The constantly developing findings and the emerging COVID-19 pandemic meant that the focus of GOFG changed from one of signposting and diversionary activities to a more individually tailored project that concentrated upon individual harms and personal risk. A large majority of G&YW experienced their problems as having improved since being involved with GOFG. This is highlighted below in the case study (Figure 1).

### Restarting the Conversation

While some conversations are best left, we really do need to start talking again about vulnerable G&YW. Especially considering the most recent Crime Survey in England & Wales^3^ (2021) which estimates that in the year ending March 2020, 618,000 women have experienced sexual assault (HM Government, 2021). In light of these figures and the political landscape since the Sarah Everard case, the impact of male violence on women and girls in the United Kingdom continues unabated. This article, while presenting emerging findings regarding interventions that can work toward helping stem the tide of community violence associated with girls and gangs, is actually describing much more than that. This article is a call to action to point out where we are after a decade of talking, funding, and skirting the edges of SYV and gang violence and how cyclical conversations continue to impact young women in a direct and non-direct way. Our work has given G&YW a voice to describe how they are experiencing their worlds today.

Directly, GOFG can evidence that girls valued their mentors and experienced the process as positive. Mentoring was delivered outside of the criminal justice system and did not stray into the domain of being “too much of a good thing” (Wong & Horan, 2021) in balancing support to an individual's agency and avoiding dependency. GOFG dosage was experienced well by the young women. There is also emerging evidence to suggest that high-intensity exercise (boxing) can have a positive impact on anxiety and depression (Jump & Blakemore, 2022), and the combination of the two worked well. So much so, that during the COVID-19 pandemic, mentoring and boxing became the only two of the offered GOFG activities that the young women engaged with (during the Pandemic G&YW were also offered alternative activities including arts and culture and health and beauty). The qualitative evidence in particular, also suggests that young women experienced the GOFG project as a psychological “safe space,” in other words, a place that allowed them to be young women with independence and aspirations.

Indirectly, the mentors were viewed as trusted adults in the process, and the young women started to slowly hear their own voice being listened to and recognize their own agency, arguably building upon the humanizing aspect of Ansbro's (2022) work into aforementioned attachment theory and probation practices. Moreover, recent evidence by Buck (2020) suggests that peer mentors are well placed to act as inspirational role models and can offer reassurances that change is manageable and positive futures are possible.

All this combined, while considering the historical work of Firmin (2009), Young (2009), and Batchelor (2005, 2009), these small elements of change are big strides for young women who prior to any form of intervention were struggling with high levels of need and had nowhere to turn. Contextual safeguarding in particular is attempting to address these needs as is the nascent area of Transitional Safeguarding by recognizing the manifold of social contexts within which the harm can occur. Yet, we found that by prioritizing individual agency coupled with mental health support we were able to target the high level of presenting need among a cohort of over 100 young women, while slowly developing trust within the mentoring relationship to inspire personal change among the G&YW.

## Discussion

We began this article by suggesting that we need to restart the conversation of SYV, gangs, and the impact of these things on women and girls. When Firmin (2009) and others were writing over a decade ago they were passionately arguing for change, and a recognition of the harms that SYV and gangs were perpetrating against females in communities ravaged by these factors. Now, as we write in 2024, small strides have been made with projects such as GOFG, and the policy landscape is arguably more poised than ever to be seen to being addressing these harms (Jump & Horan, 2021). The learning emerging from GOFG builds upon wider research and evidence that shows how gang-involved or affected G&YW have to navigate a range of harmful environments which can expose them to high levels of sexual exploitation and increased criminal activity (Eshalomi, 2020; Firmin, 2009; Hughes et al., 2015; Jump et al., 2023). This article therefore contributes to an understanding of the context and situation of G&YW who are gang affected, and how interventions can be designed and implemented to respond to this burgeoning issue. Instead of focusing, or extrapolating evidence from interventions and approaches with young men, GOFG has worked directly with G&YW who are impacted by SYV and gangs. By working with the presenting needs of young women and involving them in the process, GOFG was able to work within a psychosocial framework with an overall aim of reducing harm to G&YW through building their individual agency and social capital. We look forward to presenting the findings from the second phase of GOFG and understanding longitudinal data over a 5-year period.

The Centre for Mental Health (Khan et al., 2013) report identified a host of preventative measures that could be taken to assist in the process, and these were included in the design and intervention strategy of the GOFG project. For example, strengthening girls’ self-esteem and responding quickly to the first signs of mental ill health among children. We conclude by reflecting upon our learning and its contributions toward understanding and responses toward VAWG. In our previous article (Jump & Horan, 2021), we identify how GOFG has scraped the surface of G&YW's presenting issues, such as neglect, care experience, school exclusion, drugs and alcohol misuse, and mental health and emotional needs, that are concomitant with VAWG. By building upon the previous tried and tested approaches and working within a more safeguarding and child-protective perspective, GOFG has recognized the vulnerability of G&YW early enough to make meaningful change. As discussed by the (HM Government, 2021; Home Affairs Select Committee, 2020) our results support the amalgamation of safeguarding and mental health responses. We suggest that gang-involved or affected G&YW benefit from holistic support that responds on an individual and contextual level at all levels of approach from primary universal intervention through to tertiary response, building agency, developing emotional well-being, and responding to their contextual environment.

The most salient question now is whether we are further on in addressing the harms caused to females as a result of serious youth and community violence? Or do we, as academics and practitioners, still need to consider the changing landscape of austerity, adolescent mental health, and childhood trauma a decade on from Firmin?

### Next Steps

In answer to the above questions, GOFG made a series of recommendations that addressed the issues of VAWG and the intersection of this with other presenting harms such as adolescent mental health, austerity, and childhood trauma. We advocate for a clear separation from the wider discourses surrounding gang prevention and youth justice and a recognition of the vulnerability of exploited G&YW early enough to make meaningful change. Importantly, we recommend that VAWG needs to be acknowledged within this context. We observe that VAWG is not separate to girls at risk of, or involved in SYV, gangs, and related vulnerabilities. Response needs to be integrated. It is a Venn diagram; they are not mutually exclusive. Put simply, the needs of G&YW differ from those of young men. With this recognition, those working in the field can develop young women's autonomy and agency to become the authors of their own journeys. Through good mentorship, young women can be enabled and supported toward their goals, which increases their individual agency to make meaningful change and realize their goals. Moreover, mental and emotional health support is critical to building this agency and capital, and therefore, the amalgamation of safeguarding and mental health responses is key to addressing G&YW's needs. We suggest that targeted and expedited mental health intervention enhances protective factors surrounding Child Sexual Exploitation and Child Criminal Exploitation and this was witnessed throughout the trajectory of the GOFG project.

Lastly, Firmin (2009) argued that the impact of SYV- and gang-related violence was a child protection issue and therefore should be recognized as such in both policy and practice. This has been developed in Contextual Safeguarding approaches discussed earlier; however, the recommendations made by Firmin, especially regarding the subsequent planning and policy ratification needed at both governmental and local levels to overhaul and consider VAWG as a serious issue are only just being addressed. The funding needed to design and develop specialist provision that meets the needs of vulnerable young women can be patchy, and accordingly, the austerity measures in 2023/24 arguably only exacerbate this issue. It is our hope that a decade on from this article the terrain will look different, and G&YW will not be marginalized in the political and policy landscape that arguably only views this issue as an appendage to the male criminological discourse in which it resides.

## Notes

1. The GOFG theory of change formulation is presented by the authors in a separate paper.

2. Mentalisation helps individuals make sense of their own thoughts, wishes, beliefs and is particularly useful for people who suffer from long-term difficulties in relationships and who experience intense emotional distress with overwhelming feelings that may result in destructive behaviors such as self-harm or aggression toward others.

3. CSEW provides a good reflection of the true prevalence of crime (including where it is not reported to the police).
